# Identification of high-affinity Monoamine oxidase B inhibitors for depression and Parkinson’s disease treatment: bioinformatic approach of drug repurposing

**DOI:** 10.3389/fphar.2024.1422080

**Published:** 2024-10-09

**Authors:** Moyad Shahwan, Pratibha Prasad, Dharmendra Kumar Yadav, Nojood Altwaijry, Mohd Shahnawaz Khan, Anas Shamsi

**Affiliations:** ^1^ Center for Medical and Bio-Allied Health Sciences Research, Ajman University, Ajman, United Arab Emirates; ^2^ Department of Clinical Sciences, College of Pharmacy and Health Sciences, Ajman University, Ajman, United Arab Emirates; ^3^ Basic Medical and Dental Sciences Department, College of Dentistry, Ajman University, Ajman, United Arab Emirates; ^4^ Gachon Institute of Pharmaceutical Science and Department of Pharmacy, College of Pharmacy, Gachon University, Seongnam, Republic of Korea; ^5^ Department of Biochemistry, College of Science, King Saud University, Riyadh, Saudi Arabia

**Keywords:** Monoamine oxidase B, drug repurposing, virtual screening, molecular dynamic simulation (MD), molecular docking

## Abstract

Depression and Parkinson’s disease (PD) are devastating psychiatric and neurological disorders that require the development of novel therapeutic interventions. Drug repurposing targeting predefined pharmacological targets is a widely use approach in modern drug discovery. Monoamine oxidase B (MAO-B) is a critical protein implicated in Depression and PD. In this study, we undertook a systematic exploration of repurposed drugs as potential inhibitors of MAO-B. Exploring a library of 3,648 commercially available drug molecules, we conducted virtual screening using a molecular docking approach to target the MAO-B binding pocket. Two promising drug molecules, Brexpiprazole and Trifluperidol, were identified based on their exceptional binding potential and drug profiling. Subsequently, all-atom molecular dynamics (MD) simulations were performed on the MAO-B-ligand complexes for a trajectory of 300 nanoseconds (ns). Simulation results demonstrated that the binding of Brexpiprazole and Trifluperidol induced only minor structural alterations in MAO-B and showed significant stabilization throughout the simulation trajectory. Overall, the finding suggests that Brexpiprazole and Trifluperidol exhibit strong potential as repurposed inhibitors of MAO-B that might be explored further in experimental investigations for the development of targeted therapies for depression and PD.

## 1 Introduction

Depression and Parkinson’s disease (PD) represent two formidable challenges in the field of psychiatric and neurological disorders. These diseases affect millions of individuals worldwide and place a substantial burden on both healthcare systems and society at large (Van Bulck, Sierra-Magro, Alarcon-Gil, Perez-Castillo & Morales-Garcia, 2019; Su et al., 2021). These conditions share a common underlying pathophysiology marked by the dysregulation of monoamine neurotransmitters, particularly dopamine, and serotonin (Mayeux, Stern, Sano, Williams & Cote, 1988). Monoamine oxidases (MAOs) are a family of flavin-containing enzymes localized to the outer mitochondrial membrane (Iacovino et al., 2018). Among the various enzymes implicated in the catabolism of these neurotransmitters, Monoamine Oxidase B (MAO-B) has emerged as an important molecular player (Huang, Chen, Loh, Chan & Hong, 2021). MAO-A and MAO-B, two prominent isoforms of this protein family, exhibit distinct substrate specificities (Finberg and Rabey, 2016).

MAO-A preferentially metabolizes serotonin, norepinephrine, and dopamine, while MAO-B primarily targets phenylethylamine and benzylamine (Finberg, 2014). However, MAO-B plays a pivotal role in the oxidative deamination of dopamine which makes it a promising target for therapeutic intervention in both depression and PD (Duarte et al., 2021). Dopamine is a key neurotransmitter in the brain’s reward and pleasure systems and plays a critical role in the pathogenesis of depression (Dunlop and Nemeroff, 2007). Dysregulation of dopaminergic pathways has been implicated in anhedonia and motivational deficits characteristic of depressive disorders (Belujon and Grace, 2017). Understanding the role of dopamine is crucial for the development of specific pharmacological interventions for depression (Dunlop and Nemeroff, 2007). Since MAO-B reduces dopamine levels, its inhibitors may help to prevent the dopaminergic deficits that underlie these disorders (Finberg, 2010). Thus, MAO-B inhibitors are considered as potential drugs with a two-fold effect in treating depression and neurological disorders such as PD (Finberg and Rabey, 2016). The MAO-B inhibition also reduces oxidative stress, which is linked to various neurodegenerative disorders and other health issues (Marconi et al., 2019).

Depression remains a leading cause of disability worldwide (Friedrich, 2017). While the exact etiology of depression is not yet fully elucidated, evidence implicates a deficiency in serotonin and norepinephrine neurotransmission (McGrath, Baskerville, Rogero & Castell, 2022). Traditional antidepressant medications, such as selective serotonin reuptake inhibitors (SSRIs) and serotonin-norepinephrine reuptake inhibitors (SNRIs), have been the primary pharmacological interventions in treating depression (Sansone and Sansone, 2014). However, these drugs are not universally effective, and many patients do not achieve standard treatments (Danborg et al., 2019). Moreover, the onset of therapeutic action with these agents typically takes several weeks (Kroll, 2022). Consequently, PD is characterized by motor symptoms, such as bradykinesia, resting tremor, rigidity, and postural instability, which result from the progressive degeneration of dopaminergic neurons in the substantia nigra (Balestrino and Schapira, 2020). The standard therapeutic in PD primarily involves the use of levodopa, a precursor to dopamine which temporarily alleviates motor symptoms (Emamzadeh and Surguchov, 2018). Moreover, long-term levodopa treatment can lead to motor fluctuations and dyskinesias (Thanvi and Lo, 2004). Consequently, there is a pressing need for therapies to address the motor symptoms with neuroprotection for slowing down the disease progression.

Here, in this study, we chose to focus on MAO-B rather than MAO-A as its activity increases with age (Fowler et al., 1997). In contrast to MAO-A, which exhibits moderate changes in activity throughout the life span, MAO-B activity increases with age and gliosis and thus increases dopamine degradation (Saura et al., 1997). MAO-B is more selective; therefore, it is possible to achieve a more specific therapeutic intervention aimed at maintaining dopaminergic function in these groups of patients (Finberg and Rabey, 2016). By therapeutic targeting of MAO-B, it is possible to modulate dopamine levels that offer a dual therapeutic benefit for depression and PD (Parambi, 2020). Drug repurposing offers a unique advantage by bypassing many of the time-consuming and costly steps associated with traditional drug discovery and development (Hodos, Kidd, Khader, Readhead & Dudley, 2016). In this study, we explored the potential MAO-B inhibitors among FDA-approved drugs while exploiting a rational and systematic approach integrating molecular docking and molecular dynamics (MD) simulation (Pushpakom et al., 2019). The choice to focus on FDA-approved drugs as potential MAO-B inhibitors is guided by the wealth of clinical data available for these molecules, including safety profiles and established pharmacokinetics (Cha et al., 2018). This repurposing approach minimizes the risk associated with early-phase drug development which offers a pragmatic and efficient strategy for drug discovery (Park, 2019). This study showcases the promise of computational drug repurposing and emphasizes the significance of MAO-B as a target for depression and PD therapies. The repurposing of FDA-approved drugs as high-affinity MAO-B inhibitors could herald a new era in the treatment of depression and PD.

## 2 Materials and methods

### 2.1 Receptor preparation

The crystal structure of the MAO-B was downloaded from the RCSB Protein Data Bank (PDB) with the PDB ID: 6FW0 (Reis et al., 2018). The original protein structure had a resolution of 1.60 Å and was determined through X-ray diffraction. This structure was subjected a refinement using the graphical user interface-based AutoDock Tools (Huey et al., 2012). Protonation and energy minimization were made to the protein structure to optimize it for docking and simulation studies. Further, the assignment of Kollman atom charges, the addition of polar hydrogen atoms, and the inclusion of solvation parameters were done. This structure served as the foundation for docking and simulation studies.

### 2.2 Compounds library preparation

The repurposed drug library was sourced from the DrugBank database. DrugBank is an openly accessible repository that houses a diverse collection of commercially available molecules tailored for virtual screening and pharmaceutical research (Wishart et al., 2018). This dataset comprises a total of 3,648 drug molecules in the three-dimensional Structure Data File (SDF) format. These molecules were subsequently transformed into the PDBQT format for molecular docking studies, using the Open Babel converter (O'Boyle et al., 2011). This selection and preparation of ligands from the DrugBank formed the cornerstone of our subsequent molecular docking screening.

### 2.3 Molecular docking screening

Molecular docking is a commonly employed computational technique for the prediction of the most favorable binding configuration and strength of interaction between small molecules to a protein receptor (Naqvi, Mohammad, Hasan & Hassan, 2018). In this study, molecular docking served as the primary approach for identifying the most favorable conformational poses and binding affinities of various molecules towards MAO-B. To facilitate protein-ligand docking, we utilized AutoDock Vina software with various custom-made Perl scripts (Trott and Olson, 2010). A structurally blind search space was defined, centered at coordinates X:53.393 Å, Y:147.93 Å, and Z:24.589 Å, positioned with sizes X:69 Å, Y:89 Å, and Z:84 Å. This grid space was large enough to encompass all heavy atoms of the protein. This search space allowed ligands the flexibility to explore and locate their preferential binding sites within the MAO-B structure. After the docking, molecules with superior docking scores were identified and fetched out to a sub-directory. To gain a detailed understanding of molecular interactions of screened compounds and MAO-B, all potential docked conformers were generated.

### 2.4 Biological activities and drug profiling

After filtering molecules based on their binding profiles with MAO-B, the selected hits were evaluated for their potential biological activities. For this assessment, we harnessed the power of the PASS (Prediction of Activity Spectra for Substances) online server (Lagunin, Stepanchikova, Filimonov & Poroikov, 2000). PASS is an online tool that leverages the structural formulas of molecules to forecast their probable biological activities (Lagunin et al., 2000). This server offers estimates of the likelihood of molecules exhibiting specific activities, providing results in two pivotal parameters: ‘Pa, probability to be active’ and ‘Pi, probability to be inactive’. A compound with a higher ‘Pa’ value signifies a greater likelihood of possessing a specific biological property. The ‘Pi’ represents the probability that a compound will be inactive for a particular biological activity. The utilization of PASS enabled us to prioritize molecules with promising therapeutic potential for further investigation and analysis.

### 2.5 Interaction analysis

To gain deeper insights into the molecular interactions involved in the ligand binding, we conducted a detailed analysis of the bonds and forces involved in the ligand-MAO-B interactions. This analysis was pivotal in elucidating the intricate nature of these interactions. For this purpose, we employed PyMOL 3.0 (DeLano, 2002) and BIOVIA Discovery Studio Visualizer 2023 (Visualizer 2005). These software tools allowed us to generate detailed docking output representations for the filtered molecules with the MAO-B binding site. After the generation of these visualizations, we specifically focused on those molecules that exhibited key interactions with important residues located within the MAO-B binding site.

### 2.6 MD simulations

MD simulations play a crucial role in drug discovery to understand the binding mechanisms and dynamics of potential drug candidates within their target proteins. To gain deeper insights into the dynamic behavior of the selected molecules with MAO-B, we conducted MD simulations using GROMACS 2020 beta (Van Der Spoel et al., 2005). The starting structures for these simulations were derived from the MAO-B-compound complexes obtained from the docking study. To ensure the accurate representation of the protein-ligand interactions, we solvated these complexes in a cubic box filled with the SPC216 model (Glättli et al., 2002). This cubic box extended at least 10 Å away from the protein surface in all directions. The system was further neutralized by the addition of counter ions (Na^+^ and Cl^−^) to achieve a concentration of 0.15 M. Energy minimization was carried out using the steepest decent algorithm. The equilibration steps were performed for 1,000 picoseconds (ps) of NVT and NPT simulations. The production run was conducted for 300 ns with a time step of 2 femtoseconds (fs). The MD simulation trajectories were analyzed using the GROMACS software package. Multiple tools in this package provide valuable insights into the dynamic behavior and stability of the protein-ligand interactions over an extended time scale. To visualize and interpret the MD simulation results, we utilized VMD (Visual Molecular Dynamics) software.

## 3 Results and discussion

### 3.1 Molecular docking analysis

Molecular docking screening is a widely used approach to identify potential small molecule ligands that can bind to a target protein with high affinity and specificity. To identify potent binders of MAO-B with exceptional binding affinities, we exploited the power of structure-based molecular docking. The study results in the identification of several molecules with notable binding affinities to MAO-B. From the 3,648 molecules docked to MAO-B, we elucidated the top 10 hits based on their binding affinity. These selected molecules exhibited binding affinities ranging from −11.2 to −12.1 kcal/mol concerning MAO-B (Table 1). The findings emphasized a direct correlation between lower binding energy and increased inhibitory potential. All the selected compounds showed higher affinity than the reference inhibitor of MAO-B, Chlorophenyl-chromone-carboxamide (∼{N}s-(3-chlorophenyl)-4-oxidanylidene-chromene-3-carboxamide) (Reis et al., 2018). The higher binding efficiency observed in the selected molecules points toward their promising prospects as high-affinity inhibitors of MAO-B. This initial screening laid the foundation for a more in-depth investigation of these potential inhibitors in the subsequent stages of our study.

**TABLE 1:** Binding affinity and other docking parameters of the selected hits from the FDA library. pKi represents the dissociation constant.

S. No.	Drug	Binding affinity (kcal/mol)	pKi	Ligand efficiency (kcal/mol/non-H atom)	Torsional energy
1	Risperidone	−12.1	8.87	0.4033	1.2452
2	Aminoquinuride	−12.0	8.80	0.4286	1.2452
3	Bagrosin	−11.9	8.73	0.5409	0.3113
4	Talniflumate	−11.5	8.43	0.3833	1.8678
5	Brexpiprazole	−11.5	8.43	0.3710	2.1791
6	Doxazosin	−11.4	8.36	0.3455	1.5565
7	Perflunafene	−11.3	8.29	0.4036	0
8	Nafamostat	−11.3	8.29	0.4346	1.8678
9	Tedizolid Phosphate	−11.3	8.29	0.3645	2.4904
10	Trifluperidol	−11.2	8.21	0.3862	2.4904
11	Chlorophenyl-chromone-carboxamide	−7.8	5.72	0.3714	0.6226

### 3.2 Biological properties and drug profiles

When designing effective and safe drugs, it becomes imperative to estimate the range of biological activities and potential targets of the studied molecules. We have performed PASS analysis to explore the drug profiles and biological activities of the screened compounds (Supplementary Table S1). Here, based on the drug profiling and promising biological activities, two molecules, Brexpiprazole and Trifluperidol were selected from the PASS analysis (Table 2). The results revealed that these molecules exhibited substantial potential across various biological activities. They displayed maximum Pa values ranging from 0.303 to 0.854, signifying a strong likelihood of Antineurotic, Antipsychotic, Antiparkinsonian, and Antidepressant properties. Another valuable consideration in the pharmacokinetic analysis is the blood-brain barrier (BBB) permeability. The results showed that both the selected molecules are BBB permeable. The molecular docking analysis of Brexpiprazole and Trifluperidol with MAO-A showed that they have lower binding affinities (−8.7 to −8.6 kcal/mol, respectively) than MAO-B. This observation underscores the versatility and potential multifaceted applications of the selected molecules towards MAO-B inhibition for therapeutic development against depression and neurological disorders such as PD. Given their notable biological properties, these molecules were deemed especially promising and merited further investigation. They were subsequently chosen for in-depth interaction analysis, as outlined in the subsequent sections.

**TABLE 2:** PASS biological properties of the selected hits from screening of the FDA library.

Drug	BBB permeability	Biological activity	Pa	Pi
Brexpiprazole	Permeable	Antineurotic	0.729	0.033
		Acute neurologic disorders treatment	0.620	0.033
		Antipsychotic	0.409	0.031
		Mood disorders treatment	0.377	0.039
		Antidepressant	0.368	0.040
Trifluperidol	Permeable	Antineurotic	0.854	0.009
		Antiischemic, cerebral	0.743	0.021
		Antipsychotic	0.548	0.016
		Antiparkinsonian, rigidity relieving	0.363	0.031
		Antidepressant, Imipramin-like	0.303	0.019
Chlorophenyl-chromone-carboxamide	Permeable	5-O-(4-coumaroyl)-D-quinate 3′-monooxygenase inhibitor	0.428	0.123
		MAO inhibitor	0.362	0.005
		MAO B inhibitor	0.272	0.005
		MAO A inhibitor	0.099	0.020
		Antiparkinsonian	0.211	0.145

### 3.3 Interaction analysis

**FIGURE 1:**
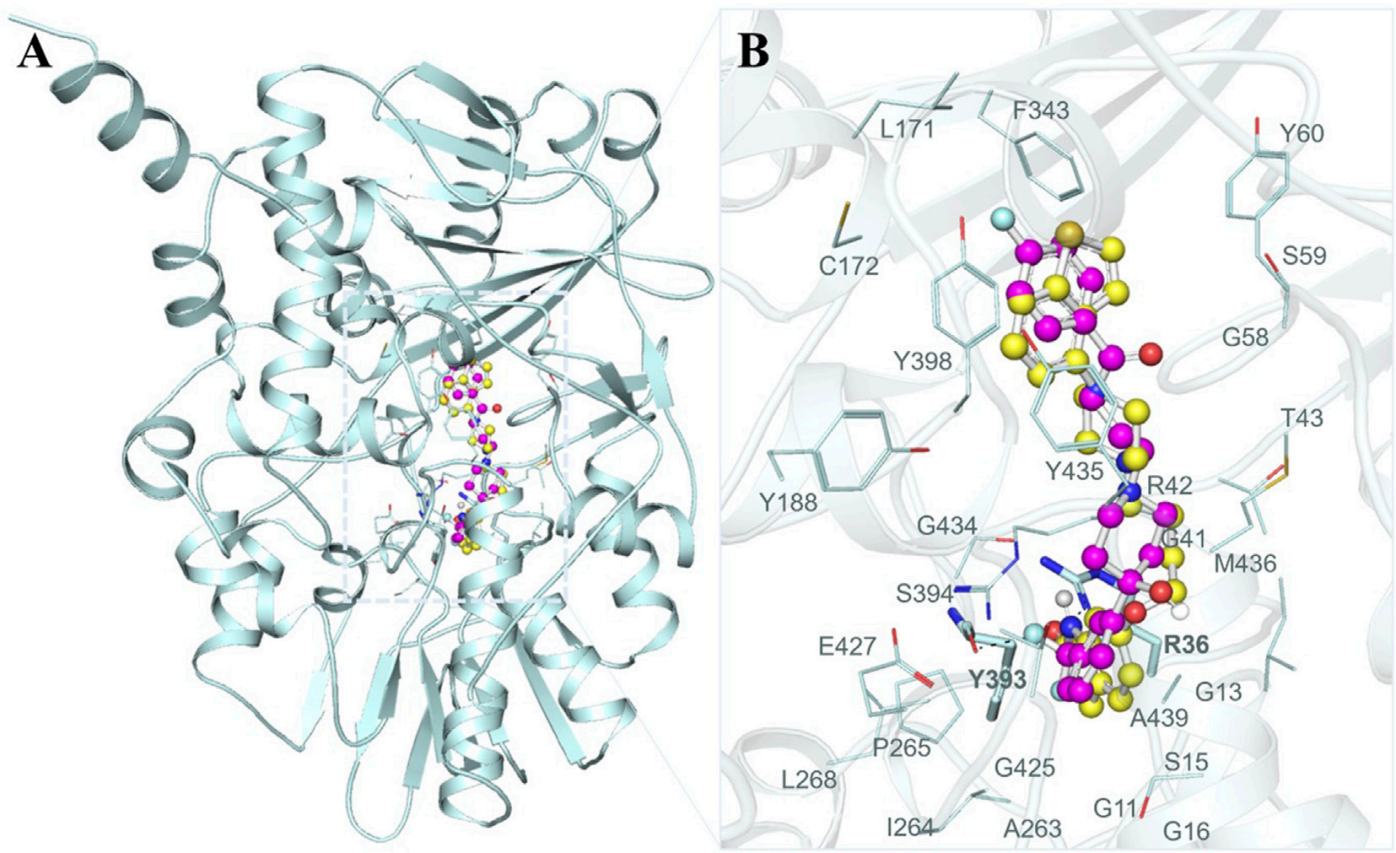
Structural depiction of MAO-B with Brexpiprazole and Trifluperidol **(A)** Cartoon representation of MAO-B with the selected molecules. **(B)** The magnified view of the interaction of MAO-B with Brexpiprazole and Trifluperidol.

To gain a profound understanding of the specific interactions between the two selected molecules, Brexpiprazole and Trifluperidol, and the binding site of MAO-B, we performed a detailed interaction analysis. This analysis involved the extraction of all potential docking conformations from their respective output files. The interaction patterns between the molecules and MAO-B were examined using PyMOL 3.0 and Discovery Studio Visualizer 2023. As illustrated in Figure 1A, our investigation primarily focused on the interaction of Brexpiprazole and Trifluperidol, with a set of important residues located within the MAO-B binding pocket. A closer examination, as depicted in Figure 1B, offered an expanded view of the protein-ligand interactions. This analysis unveiled a particularly significant interaction with many important residues critically essential for the MAO-B functionality. This interaction underscored the pivotal role of these molecules as potential inhibitors.

**FIGURE 2:**
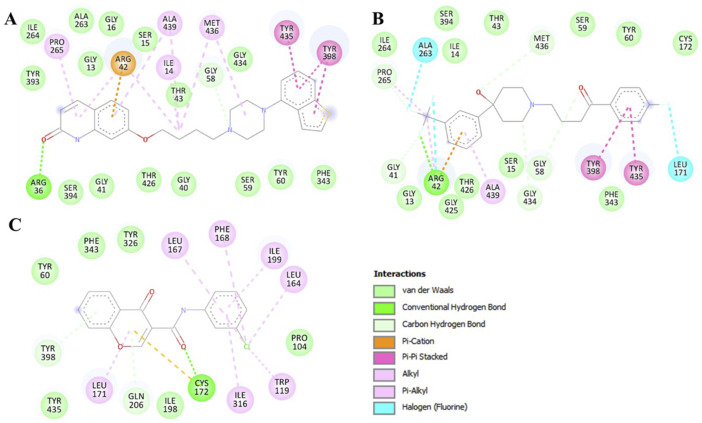
Two-dimensional depiction of MAO-B amino acid residues and their interactions with **(A)** Brexpiprazole, **(B)** Trifluperidol, and **(C)** Chlorophenyl-chromone-carboxamide.

To get a clear picture of the interaction dynamics, we also analyzed the binding of both compounds in the MAO-B binding pocket. This analysis helps to understand the critical interactions with the important residues of the protein. It is useful to gain further understanding of their possibilities as high-affinity MAO-B inhibitors. The detailed description of these interactions proved to be useful in understanding the type of interactions that occur in both complexes (Figure 2). The study brought out the fact that Brexpiprazole and Trifluperidol can dock at the active site cavity of the protein located near the FAD cofactor. These molecules form several hydrogen bonds with essential amino acids and perfectly fit the hydrophobic, flat active site of human MAO-B. The results of the present study indicate that Brexpiprazole and Trifluperidol may be repurposed MAO-B inhibitors.

### 3.4 MD simulation studies

MD simulations are useful to unravel the dynamic aspects of protein stability and protein-ligand interactions. MD simulations were used to get insights into the behavior of ligand-free MAO-B and ligand-bound MAO-B with Brexpiprazole and Trifluperidol for a 300 ns time scale. The MD analyses played a fundamental role in validating the stability and behavior of these complexes, as explored through various parameters discussed in the ensuing sections.

#### 3.4.1 Dynamics of MAO-B structure

**FIGURE 3:**
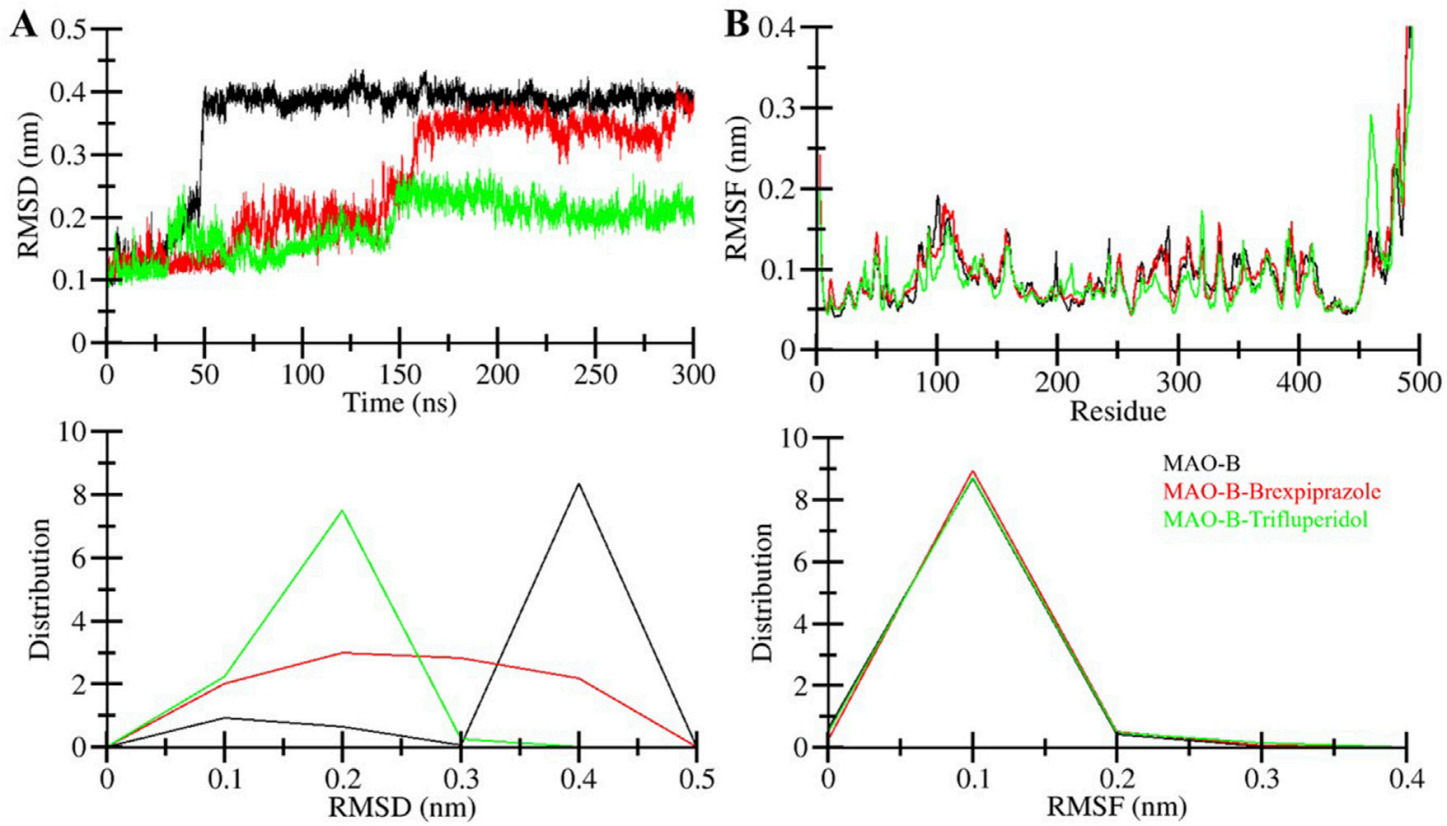
Structural dynamics of MAO-B. **(A)** RMSD graph showing MAO-B and when it is bound with elucidated molecules. **(B)** The average RMSF of MAO-B and upon Brexpiprazole and Trifluperidol binding. The lower panels show the probability distribution function of the observed values.

After the binding of small chemical molecules to a protein structure, it is common to observe structural changes and variations in its compactness (Maruyama et al., 2023). To investigate these changes and assess structural dynamics in MAO-B, we employed root-mean-square deviation (RMSD) parameter. RMSD measures the deviation of atom positions within a protein from their initial position (Pitera, 2014). The RMSD calculations were performed for all systems, MAO-B in its free and ligand-bound states. Figure 3A visualizes the deviations in the protein backbone throughout the simulations. The average RMSD values were determined between 0.1 and 0.4 nm for Free-MAO-B, MAO-B-Brexpiprazole, and MAO-B-Trifluperidol complexes. These values with decreasing RMSD are indicative of enhanced stability of MAO-B after the compound’s binding. A subtle variation in RMSD was observed in all MAO-B systems between 50 and 150 ns, showing system adjustments. After that, the RMSD remained remarkably stable throughout the 300 ns simulations indicating the complexes’ stability.

The root mean square fluctuation (RMSF) of a protein is a measure of its residual flexibility. It is calculated by assessing the distance of each residue from its average position during the simulation. To understand the residual dynamics in the MAO-B structure before and after ligand binding, we conducted an RMSF analysis from the simulated data. This analysis generated RMSF plots for each residue, as illustrated in Figure 3B. The RMSF variations remained consistently low and decreased after the binding of Brexpiprazole and Trifluperidol indicating exceptional stability and uniformity within the protein-ligand complexes. A slight increase in residual vibrations was observed after the binding of the ligands at some places suggesting some enhanced dynamics in the loop regions. Overall, the RMSF analysis emphasizes the overall stability and resilience of the protein-ligand interactions.

**FIGURE 4:**
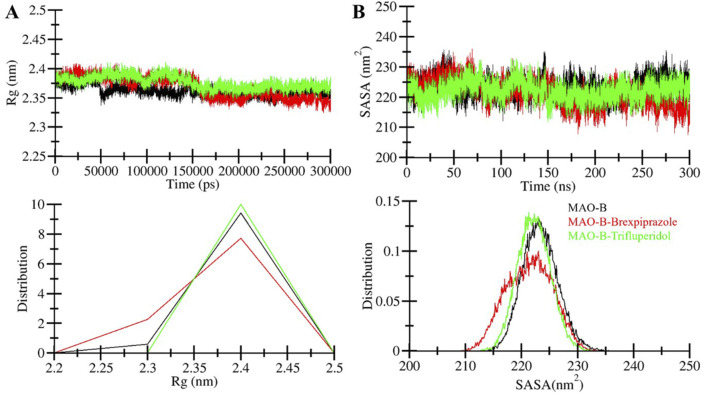
Compactness and folding of MAO-B upon binding with molecules. **(A)** The radius of gyration (*R*g) of C-alpha atoms of free protein and ligand-bound protein during simulation run through time evaluation. **(B)** SASA plot of MAO-B as a function of time before and after binding with selected molecules.

The radius of gyration (*R*g) is one of the widely used parameters in protein research that provides insights into the compactness and overall shape of the protein structure (Lobanov et al., 2008). *R*g is a valuable tool to examine the conformational behavior of a protein, such as those induced by ligand binding or environmental conditions. Here, we assessed the stability of MAO-B, MAO-B-Brexpiprazole and MAO-B-Trifluperidol complex by calculating their *R*g values in MD simulations (Figure 4A). The average *R*g for these systems was determined as 2.36 nm for free MAO-B, 2.37 nm for MAO-B-Brexpiprazole, and 2.38 nm for MAO-B-Trifluperidol. The analysis suggests that the *R*g values remained relatively stable in all three systems and maintained their structural stability and conformation.

The solvent-accessible surface area (SASA) is the area of a molecule that is accessible to solvent molecules (Marsh and Teichmann, 2011). We calculated the average SASA values for MAO-B and the MAO-B-compound complexes from the simulated trajectories. This analysis offered insights into how the presence of ligands may influence the SASA and overall conformational stability of a protein. When analyzing the SASA plots for the MAO-B in complex with Brexpiprazole and Trifluperidol, we observed consistent SASA values throughout the simulation duration (Figure 4B). This observation suggests that the MAO-B-ligand complexes retained their structural stability. Across all systems, the distribution of SASA values exhibited a consistent pattern, except for the MAO-B-Brexpiprazole complex. The MAO-B-Brexpiprazole complex demonstrated a modest decrease in the average SASA. This decrease implies a higher compact packing of the protein, particularly in the presence of Brexpiprazole bound to MAO-B. Importantly, this observation is correlated with the *R*g, suggesting that the overall protein folding remained consistent. Notably, the SASA indicates that MAO-B maintained its structural folding before and after ligand interaction, reaffirming the protein’s stability and conformational integrity.

#### 3.4.2 Secondary structure analysis

**FIGURE 5:**
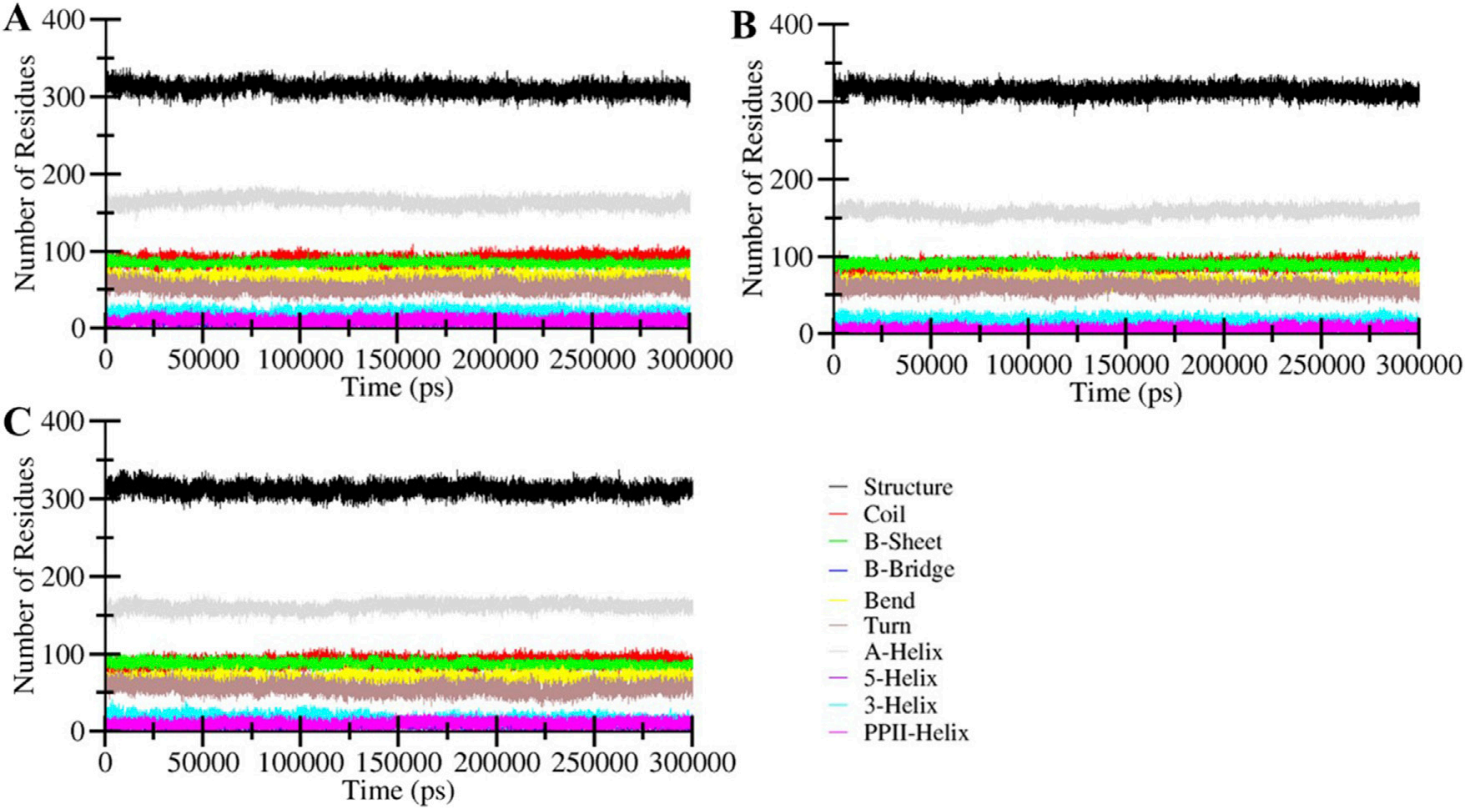
Secondary structure content of **(A)** MAO-B, and its complexes with **(B)** Brexpiprazole and **(C)** Trifluperidol.

Analysis of secondary structure elements in a protein is commonly employed to evaluate the conformational arrangements of amino acid residues within a protein (Mizuguchi and Gö, 1995). This analysis yields valuable insights into protein folding, stability, and how the binding of ligands influences its conformation. Monitoring alterations in secondary structure over time is vital to confirm the establishment of a durable complex between the protein and ligand. The secondary structure dynamic analysis signifies that the general components of the free MAO-B and the ligand-bound MAO-B complexes remained constant throughout the simulations (Figure 5). No structural shift was observed in any systems throughout the simulated trajectories. This analysis further underscores the stability of the protein-ligand complexes and the consistent arrangement of their secondary structure elements.

#### 3.4.3 Hydrogen bonding

**FIGURE 6:**
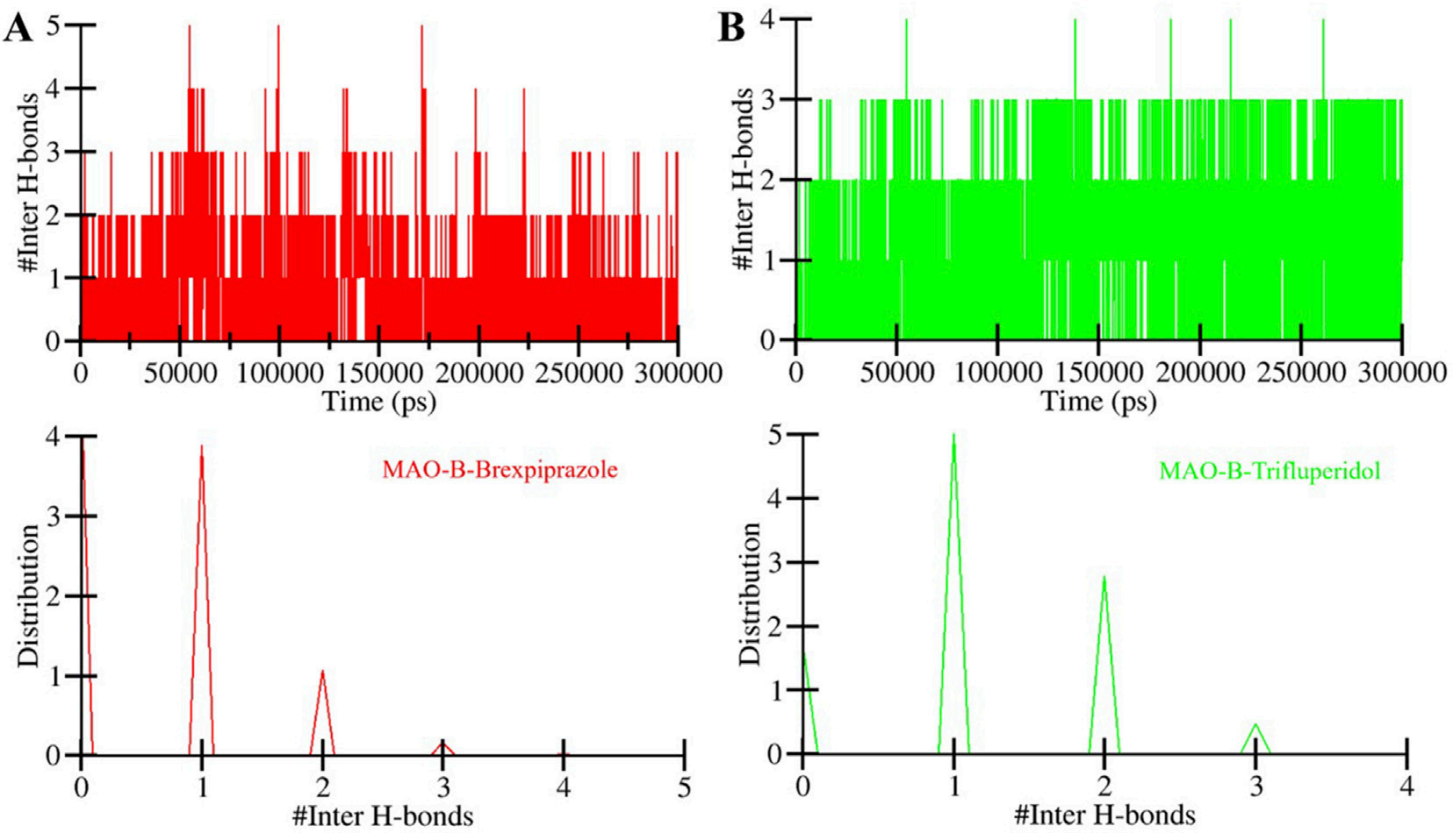
The time evolution of intermolecular hydrogen bonds formed between MAO-B and **(A)** Brexpiprazole and **(B)** Trifluperidol.

The directionality and stability of a protein-ligand complex depend on the formation and strength of hydrogen bonds between the two coordinates (Menéndez, Accordino, Gerbino & Appignanesi, 2016). These hydrogen bonds play a central role in determining drug specificity and stability (Yunta, 2017). We performed intermolecular hydrogen bond analysis of MAO-B and their interactions with the screened compounds (Figure 6). The plot showed that the number of hydrogen bonds in the MAO-B-Brexpiprazole complex spanned from 1 to 5, with a stability of 2 (Figure 6A). This indicates a certain degree of variability in the stability of the ligand-protein complexes over the simulation period. Likewise, in the MAO-B-Trifluperidol complex, the number of hydrogen bonds fluctuated between 1 and 4, with higher stability of 2 (Figure 6B). Overall, the results showed that both compounds remained in their original docked position throughout the simulation period. This analysis sheds light on the fluctuations in hydrogen bond numbers over time. This information is essential for comprehending the stability of these complexes and their potential as potent MAO-B binders with high affinity.

#### 3.4.4 Principal component analysis

**FIGURE 7:**
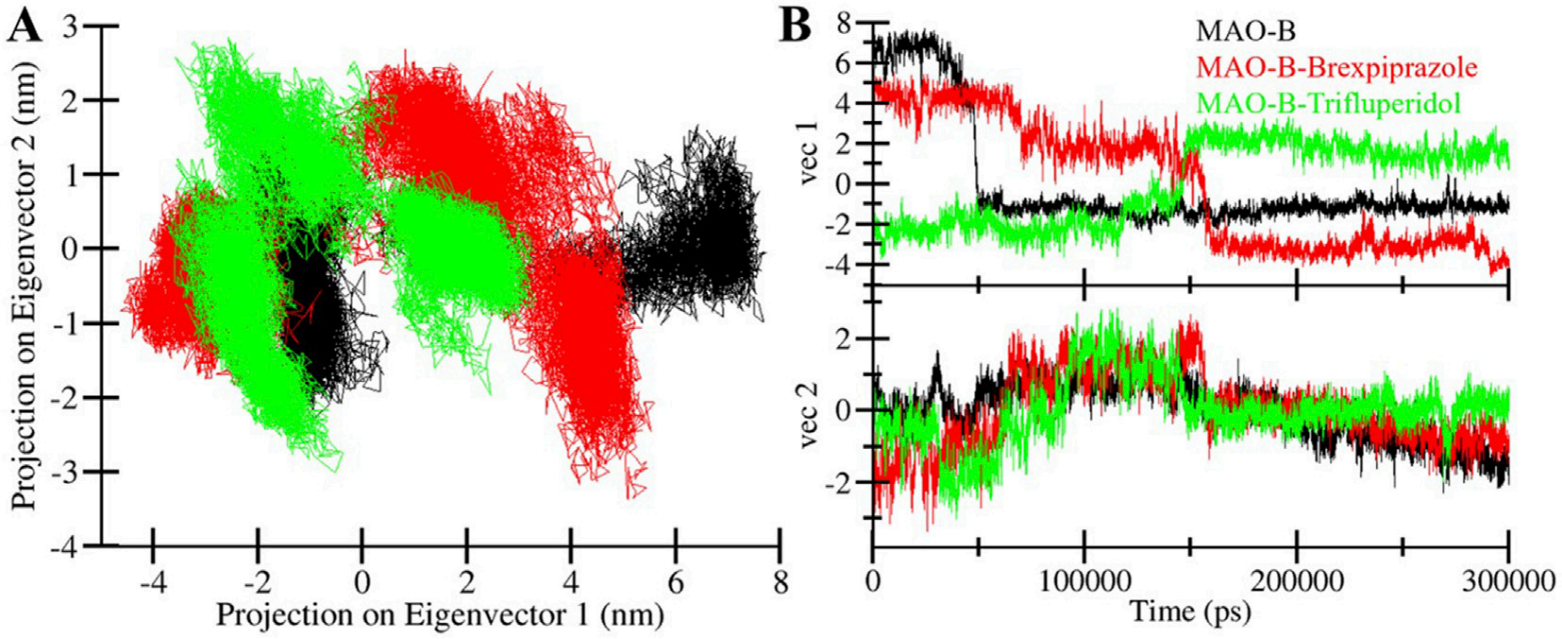
Conformational sampling in MAO-B and its docked complexes. **(A)** 2D projection of conformations landscapes and **(B)** their time evolution.

PCA is a powerful tool for exploring the conformational dynamics of proteins (Stein, Loccisano, Firestine & Evanseck, 2006). Here, we exploited PCA to explore the complex conformational changes of MAO-B, both in its unbound state and when bound to Brexpiprazole and Trifluperidol. The PCA results are presented in Figure 7 which offers the conformational landscape of these entities. Notably, we observed that the MAO-B-Brexpiprazole and MAO-B-Trifluperidol complexes largely occupy the same fundamental subspace as MAO-B in its unbound state (Figure 7A). This convergence is further supported by the Eigenvalue (EV) plots, indicating that MAO-B and the MAO-B-ligand complexes share the same phase (Figure 7B). The results indicate that the binding of Brexpiprazole and Trifluperidol has minimal impact on the protein’s conformational exploration. This alignment among the complexes highlights their structural strength and confirms the potential of Brexpiprazole and Trifluperidol as stable binders of MAO-B.

### 3.5 Free energy landscape analysis

**FIGURE 8:**
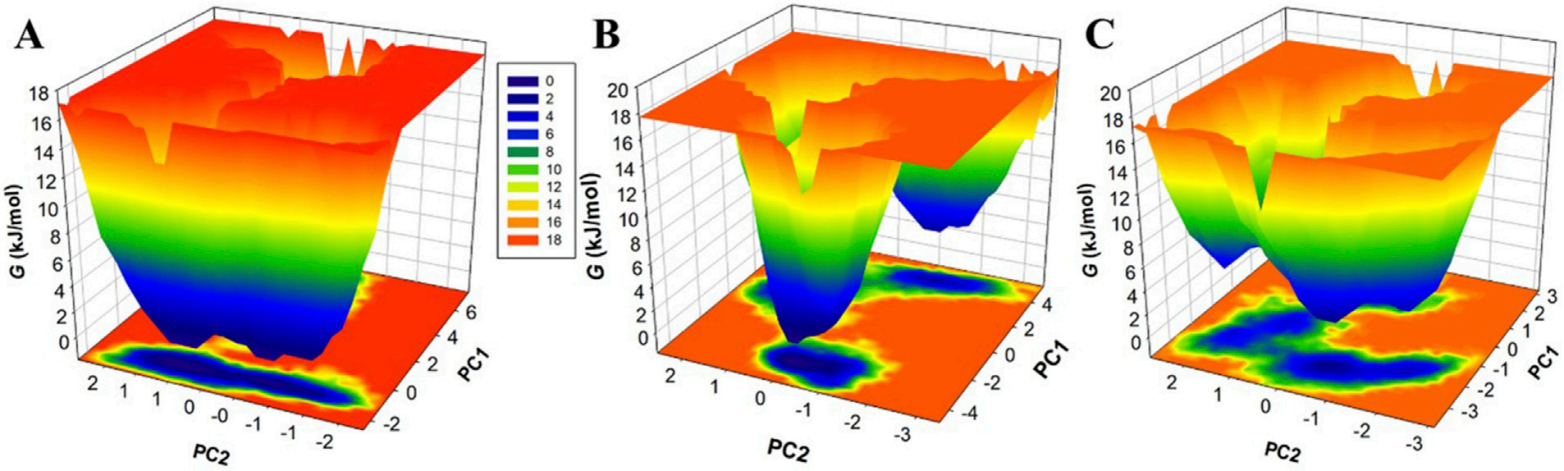
Free energy landscapes for **(A)** MAO-B, **(B)** MAO-B-Brexpiprazole, and **(C)** MAO-B-Trifluperidol.

Free energy landscapes (FELs) offer instructive visual representations of the protein folding process, leading to its native state at the global energy minimum (Papaleo, Mereghetti, Fantucci, Grandori & De Gioia, 2009). These landscapes are indispensable tools for assessing protein stability, as well as the stability of protein-ligand complexes during MD simulations (Schug and Onuchic, 2010). FELs use a color gradient to symbolize protein energy levels, with deeper blue regions indicating lower energy levels, closely approximating the native state. Here, we employed two principal components (PCs) to extract energy minima and elucidate the conformational profiles of MAO-B, MAO-B-Brexpiprazole, and MAO-B-Trifluperidol complexes. The FELs, elegantly showcased in Figure 8, provide valuable insights into changes in the size and positioning of confined phases containing 2-3 global minima when Brexpiprazole and Trifluperidol bind to MAO-B. Specifically, MAO-B predominantly occupies three global minima, extending to encompass 3 basins (Figure 8A). Similarly, MAO-B-Brexpiprazole and MAO-B-Trifluperidol also exhibit confinement to a single global minimum, characterized by 1-2 large basins (Figures 8B, C). The FEL analysis powerfully demonstrates the remarkable stability of MAO-B throughout the simulations, even in the presence of bound ligands. This valuable insight deepens our understanding of the binding mechanisms and the endurance of the identified compounds. As a result, it contributes significantly to the ongoing progress in therapeutic strategies targeting MAO-B in depression and PD.

## 4 Conclusion

In this study, we initiated an integrated search to discover potent MAO-B inhibitors that may help to mitigate the signs of depression and PD. Using molecular docking and MD simulations, we examined a comprehensive library of FDA-approved drug molecules and identified potential candidates for MAO-B inhibition. Two compounds, Brexpiprazole and Trifluperidol, have been identified to possess higher binding affinities to MAO-B than the reference inhibitor Chlorophenyl-chromone-carboxamide. By way of interaction analyses, we comprehended that these molecules built stable hydrogen bonds, hydrophobic interactions, and ionic bonding with specific residues in the MAO-B binding site. The biological activity predictions revealed that Brexpiprazole and Trifluperidol have antineurotic, antipsychotic, antiparkinsonian, and antidepressant properties. The MD simulations of the MAO-B-ligand complexes showed the structural stability of the complexes. Taken together, the present work offers a potential approach to develop novel high-affinity MAO-B inhibitors from the drugs approved by the FDA. The use of computational screening and MD simulations has provided a solid ground for further experimental confirmation and drug discovery targeting depression and PD.

## Data Availability

The original contributions presented in the study are included in the article/Supplementary Material, further inquiries can be directed to the corresponding author. Datasets are available on request.
