# Supplementary material for: Identification of high-affinity Monoamine oxidase B inhibitors for depression and Parkinson’s disease treatment: bioinformatic approach of drug repurposing
Source: Front Pharmacol. 2024 Oct 9;15:1422080. doi: 10.3389/fphar.2024.1422080 (PMC11496130; doi:10.3389/fphar.2024.1422080)
Supplement: Supplementary file 1 [file Table1.DOCX]

**Table S1**: PASS biological properties of the top 10 hits from screening of the FDA library.

| **S. No.** | **Drug molecule** | **Pa** | **Pi** | **Biological activities** |
| --- | --- | --- | --- | --- |
|  | Risperidone | 0,838 | 0,003 | 5 Hydroxytryptamine 2A antagonist |
|  |  | 0,796 | 0,018 | Nootropic |
|  |  | 0,662 | 0,004 | Alpha 1 adrenoreceptor antagonist |
|  |  | 0,607 | 0,014 | Anxiolytic |
|  |  | 0,540 | 0,005 | Alpha adrenoreceptor antagonist |
|  | Aminoquinuride | 0,785 | 0,015 | Taurine dehydrogenase inhibitor |
|  |  | 0,720 | 0,061 | Ubiquinol-cytochrome-c reductase inhibitor |
|  |  | 0,659 | 0,010 | Antiviral (Picornavirus) |
|  |  | 0,599 | 0,024 | Gastrin inhibitor |
|  |  | 0,585 | 0,016 | Platelet derived growth factor receptor kinase inhibitor |
|  | Bagrosin | 0,913 | 0,004 | Anticonvulsant |
|  |  | 0,874 | 0,010 | Testosterone 17beta-dehydrogenase (NADP+) inhibitor |
|  |  | 0,830 | 0,002 | H+-transporting two-sector ATPase inhibitor |
|  |  | 0,822 | 0,001 | CYP2C19 inducer |
|  |  | 0,793 | 0,005 | Pterin deaminase inhibitor |
|  | Talniflumate | 0,783 | 0,004 | Antipyretic |
|  |  | 0,700 | 0,003 | Chloride channel blocker |
|  |  | 0,691 | 0,026 | Fibrinolytic |
|  |  | 0,635 | 0,006 | Non-steroidal antiinflammatory agent |
|  |  | 0,618 | 0,041 | Antineoplastic |
|  | Brexpiprazole | 0,729 | 0,033 | Antineurotic |
|  |  | 0,620 | 0,033 | Acute neurologic disorders treatment |
|  |  | 0,409 | 0,031 | Antipsychotic |
|  |  | 0,377 | 0,039 | Mood disorders treatment |
|  |  | 0,368 | 0,040 | Antidepressant |
|  | Doxazosin | 0,675 | 0,004 | Alpha adrenoreceptor antagonist |
|  |  | 0,635 | 0,005 | Antiadrenergic |
|  |  | 0,632 | 0,005 | Adrenaline antagonist |
|  |  | 0,617 | 0,008 | Platelet adhesion inhibitor |
|  |  | 0,588 | 0,004 | Alpha 2c adrenoreceptor antagonist |
|  | Perflunafene | 0,947 | 0,004 | Antiinflammatory |
|  |  | 0,888 | 0,010 | Aspulvinone dimethylallyltransferase inhibitor |
|  |  | 0,872 | 0,011 | Phobic disorders treatment |
|  |  | 0,865 | 0,005 | Antiasthmatic |
|  |  | 0,853 | 0,009 | Sugar-phosphatase inhibitor |
|  | Nafamostat | 0,796 | 0,005 | Limulus clotting factor B inhibitor |
|  |  | 0,745 | 0,010 | Fibrinolytic |
|  |  | 0,722 | 0,002 | Urokinase inhibitor |
|  |  | 0,732 | 0,016 | Omptin inhibitor |
|  |  | 0,691 | 0,005 | Beta glucuronidase inhibitor |
|  | Tedizolid Phosphate | 0,787 | 0,018 | Glutamate-5-semialdehyde dehydrogenase inhibitor |
|  |  | 0,736 | 0,005 | Undecaprenyl-phosphate mannosyltransferase inhibitor |
|  |  | 0,585 | 0,014 | H+-transporting two-sector ATPase inhibitor |
|  |  | 0,572 | 0,011 | Atherosclerosis treatment |
|  |  | 0,568 | 0,028 | Trans-acenaphthene-1,2-diol dehydrogenase inhibitor |
|  | Trifluperidol | 0,854 | 0,009 | Antineurotic |
|  |  | 0,743 | 0,021 | Antiischemic, cerebral |
|  |  | 0,548 | 0,016 | Antipsychotic |
|  |  | 0,363 | 0,031 | Antiparkinsonian, rigidity relieving |
|  |  | 0,303 | 0,019 | Antidepressant, Imipramin-like |
